# Looking into the Quantification of Forensic Samples with Real-Time PCR

**DOI:** 10.3390/genes15060759

**Published:** 2024-06-09

**Authors:** Ugo Ricci, Dario Ciappi, Ilaria Carboni, Claudia Centrone, Irene Giotti, Martina Petti, Brogi Alice, Elisabetta Pelo

**Affiliations:** SOD Diagnostica Genetica, Forensic Genetic Unit, Azienda Ospedaliero-Universitaria Careggi, Largo Brambilla 3, I-50134 Florence, Italycarbonii@aou-careggi.toscana.it (I.C.); centronec@aou-careggi.toscana.it (C.C.); giottii@aou-careggi.toscana.it (I.G.); martina.petti@stud.unifi.it (M.P.); brogia@aou-careggi.toscana.it (B.A.); peloe@aou-careggi.toscana.it (E.P.)

**Keywords:** DNA amplification, polymerase chain reaction, DNA quantification, forensic DNA

## Abstract

The quantification of human DNA extracts from forensic samples plays a key role in the forensic genetics process, ensuring maximum efficiency and avoiding repeated analyses, over-amplified samples, or unnecessary examinations. In our laboratory, we use the Quantifiler^®^ Trio system to quantify DNA extracts from a wide range of samples extracted from traces (bloodstains, saliva, semen, tissues, etc.), including swabs from touched objects, which are very numerous in the forensic context. This method has been extensively used continuously for nine years, following an initial validation process, and is part of the ISO/IEC 17025 accredited method. In routine practice, based on the quantitative values determined from the extracts of each trace, we use a standard method or a low-copy-number method that involves repeating the amplification with the generation of a consensus genetic profile. Nowadays, when the quantification results are less than 0.003 ng/μL in the minimum extraction volume (40 μL), we do not proceed with the DNA extract analysis. By verifying the limits of the method, we make a conscious cost-benefit choice, in particular by using the least amount of DNA needed to obtain sufficiently robust genetic profiles appropriate for submission to the Italian DNA Forensic Database. In this work, we present a critical re-evaluation of this phase of the method, which is based on the use of standard curves obtained from the average values of the control DNA analysed in duplicate. Considering the various contributions to uncertainty that are difficult to measure, such as manual pipetting or analytical phases carried out by different operators, we have decided to thoroughly investigate the contribution of variability in the preparation of calibration curves to the final results. Thus, 757 samples from 20 independent experiments were re-evaluated using two different standards for the construction of curves, determining the quantitative differences between the two methods. The experiments also determined the parameters of the slope, Y-intercept, R^2^, and the values of the synthetic control probe to verify how these parameters can provide information on the final outcome of each analysis. The outcome of this revalidation demonstrated that it is preferable to use quantification ranges rather than exact quantitative limits before deciding how to analyse the extracts via PCR or forgoing the determination of profiles. Additionally, we present some preliminary data related to the analysis of samples that would not have been analysed based on the initial validation, from which genetic profiles were obtained after applying a concentration method to the extracts. Our goal is to improve the accredited analytical method, with a careful risk assessment as indicated by accreditation standards, ensuring that no source of evidence is lost in the reconstruction of a criminal event.

## 1. Introduction

The identification of biological traces through DNA testing is now a well-established technique worldwide. It forms the basis for the construction of DNA databases, allowing for the comparison of traces, unknown individuals, and unidentified bodies through the implementation of genetic profiles generated with the highest-quality standards [1]. The various phases of the techniques used are optimised within each laboratory, as there is no universally certified method issued by a public entity. Therefore, the reference standard is ISO/IEC 17025 [1], which generally pertains to testing laboratories. In this work, we aim to revisit in a detailed manner the phase through which the qualitative and quantitative presence of human DNA is verified in extracts from forensic samples. In every accredited forensic laboratory, the DNA present in a sample must be quantified before it progresses to STR PCR for DNA profiling [2]. The importance of this analytical phase is underscored by the early guidelines of the forensic community. In 1992, one of the first recommendations of the International Society of Forensic Haemogenetics indicated that DNA quantification is important for improving the quality of PCR-based DNA typing results. Therefore, initial quantification of human DNA should be always performed before PCR amplification [3]. Nowadays, the assumption that a trace may contain human biological material is sometimes contradicted by reality, as it is often possible to identify biological traces at crime scenes that are not of human origin [4].

The spectrophotometric measurement method was chosen because of its simplicity, quickness, low cost, and reliability [5]. Especially when samples are taken a long time after deposition, it is possible to find DNA of bacterial origin, which is co-extracted together with human DNA. It is thus preferable to use real-time PCR methods, as 260 nm spectrophotometry is not suitable for discriminating the different types of DNA. Over the years, commercially available qPCR kits, such as PowerQuant™ System (Promega, Madison, WI, USA) [6,7], QuantiFiler™ Trio (Thermo Fisher Scientific, Waltham, MA, USA) [8] or Investigator Quantiplex Pro (QIAGEN, Hilden, Germany) [9], have been made available by commercial companies. These systems use hydrolysis probes labelled with two fluorescent dyes: a reporter dye at the 5′ end and a quencher dye at the 3′ end [10]. Briefly, when the probe is intact, the two dyes are in close proximity due to the small size of the probe, and the fluorescence of the reporter dye is suppressed. During the extension stage of qPCR, the 5′-exonuclease activity of DNA polymerase displaces the bound hydrolysis probes, and the reporter dye attached to the 5′ end is separated from the quencher dye at the 3′ end. This separation means the energy transfer that suppressed fluorescence when the two fluorophores were in close proximity is no longer occurring and the reporter molecule can now fluoresce. The amount of fluorescence detected correlates directly with the amount of human DNA present in the reaction, as the greater the amount of DNA, the more reporter dye molecules are cleaved, and the greater the fluorescence detected. Typically, as the qPCR program progresses, and the DNA increases exponentially, an amplification curve is generated using these fluorescence data, which (at the end of the qPCR program) are then used to calculate a DNA concentration [2]. Measured DNA concentrations are used to normalise the amount of DNA template to an optimal range for STR amplification reactions, ensuring STR profiles are in the linear range of the capillary electrophoresis instrument with minimal artefacts and stochastic effects. Every available qPCR assay that is routinely used to determine the concentration of total human DNA (and human male DNA) is based on the construction of standard curves with DNA at known concentrations. Each point of the curves is constructed using the mean values of serial dilutions assayed in duplicate.

Our experience from 2016 to today is based on the use of the Quantifiler™ Trio DNA Quantification Kit (ThermoFisher Cat. No. 4482910) for the quantification of DNA extracts from stains [11]. The Quantifiler™ Trio DNA Quantification Kit uses multiple-copy target loci for improved detection sensitivity and combines four 5′ nuclease assays. The human-specific target loci (Small Autosomal, Large Autosomal, and Y-chromosome targets) each consist of multiple copies dispersed on various autosomal chromosomes (Small Autosomal and Large Autosomal), or multiple copies on the Y-chromosome. The two separate target-specific human assays, one with a short PCR amplicon and one with a long PCR amplicon, are able to determine the state of degradation of the sample. The fourth target (internal PCR control, IPC) is a synthetic probe capable of identifying the presence of inhibitors in extracts. This quantification method is part of the analytical process of the laboratory’s ISO/IEC17025 accredited method identified with the denomination “DNA typing for human identification, mixed stains, Y-STR, paternity and kinship testing (genetic profile)” by the Italian Accreditation Institution, Accredia (Lab nr. 1268) [12]. In the indicated period, it was used for 240 experiments for a total of more than 10,000 forensic samples. The amount of DNA in these samples can vary from tens of nanograms to a few picograms, corresponding to a few diploid genomes.

**Figure 1:**
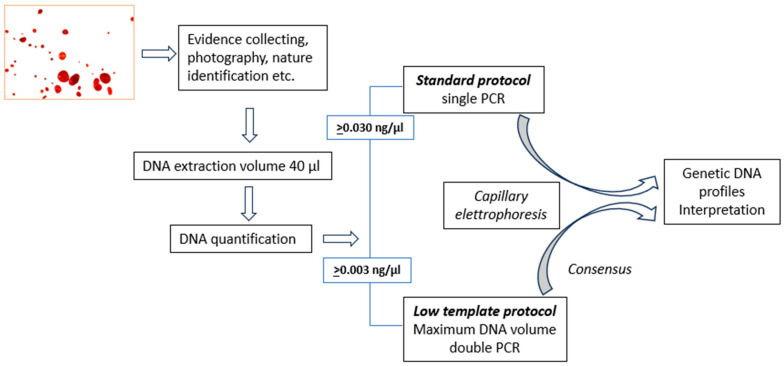
Diagrammatic representation of the forensic genetic analysis process used by the Forensic Genetic Unit of AOU Careggi. The method is accredited according to the ISO/IEC 17025 standard by the Italian Accreditation Body, Accredia (Lab nr. 1268) with the denomination “DNA typing for human identification, mixed stains, Y-STR, paternity and kinship testing (genetic profile)”.

The method we use consists of a “standard protocol” that is used when sufficient DNA is present to obtain genetic profiles from a single PCR reaction, and a so-called “low-template scenario” protocol when the amount of template DNA is low. In this case, a double amplification of the sample is performed and the results of the analysis are therefore derived from the comparison of the two replicates [13]. If the quantification result is below a certain limit, the PCR is not carried out and the outcome of the assessment is that the sample does not contain sufficient human DNA. In the initial validation process, we identified quantitative limits for applying the standard and low-template scenario protocols. A visual representation of this protocol is represented in Figure 1.

Obviously, analyses with low-template scenarios have several limitations. Indeed, allele dropout, stutter, false alleles, and the influence of sporadic background contamination should be considered when interpreting these DNA profiles [14]. However, these profiles are often found on items that have been touched by the person of interest, which are those most frequently requested by investigators for inclusion in the DNA database. At times, the only thing that allowes a person to be connected to a crime is a faint DNA trace. For this reason, it is crucial to pay utmost attention to extracting as much information as possible from these specimens. However, this activity must be balanced with a robust laboratory routine to identify an optimal cost–benefit synthesis. 

The quantitative limits mentioned above were determined through an initial laboratory validation. In DNA quantification, precision is crucial to ensure that the obtained results are reliable and consistent. Precision refers to the consistency and reproducibility of measurements obtained from repeated analyses of the same sample under the same conditions. Accuracy refers to the closeness of a measured value to the true or accepted value. However, we are aware that certain parameters can influence the final analytical results. One parameter that seems not to be evaluated with due attention is the uncertainty due to the use of measuring instruments and manual activities. This parameter is carefully evaluated in many other fields [15] and should be carefully assessed within the forensic context, as suggested by some authors [16]. Indeed, an important factor regarding the reproducibility of the standard curve is the mixing function during the pipetting of serial dilutions by the liquid-handling system. In the method used in our laboratory, quantification requires all manual steps, including the construction of the calibration curve with the dilution of the control DNA and pipetting in the sample plate. In DNA quantification, accuracy is essential to ensure that the reported DNA concentration reflects the actual amount of DNA present in the sample. ISO/IEC 17025 mandates that accredited laboratories validate the accuracy of their quantification methods by comparing their results with reference materials of known DNA concentration. Thus, the piston pipettes we used are annually calibrated by an external supplier accredited for calibrations according to Accredia. The bioanalytical approaches used by the supplier involve gravimetry according to ISO 8655 guidelines (ISO 8655-6) [17]. However, all these precautions can be affected by human random errors in the pipetting of standards and samples. Constructing a standard curve with a replicate of each control could compensate for pipetting uncertainty. Using the numerous data available, we have carried out a careful review of the individual points of the construction of calibration lines, to verify how these lines influence the final results Based on the results obtained, appropriate actions were implemented to optimise this step in the forensic genetic method we use for DNA typing. This analysis can be considered a revalidation of the method, in accordance with point 7.2.2.2 of the ISO/IEC 17025:2017 standard [18]. 

Finally, we show that, even if the results of the quantifications of DNA extracts from minimal traces are practically zero, it is possible to use a sample concentration approach, which in some cases has proven useful for generating genetic profiles of the trace donors. These indications suggest room for improvement for a technique that already provides fundamental results in the forensic genetics analysis process.

## 2. Materials and Methods

### 2.1. DNA Extraction

For automated purification of DNA from forensic casework and human samples, EZ1^®^ Advanced XL instruments in combination with the EZ1 Investigator kit were used (Qiagen, Hilde, Germany). To obtain the the highest possible DNA concentration a 40 μL final volume of TE for eluition was always used.

### 2.2. Analytical Phase of the Quantification Method

Quantitative PCR was performed using 96-well plates in a QuantStudio™ 7 Flex Real-Time PCR System (Thermo Fisher Scientific™,Waltham, MA, USA). Data analysis was performed by using Design & Analysis Software ver. 2.8.0 (Thermo Fisher Scientific™, Waltham, MA, USA).

The human DNA used to generate the DNA quantification standard dilution series consisted of pooled human male genomic DNA with concentrations ranging from 50 ng/μL (St. 1) to 0.005 ng/μL, or 5 pg/μL (St. 5). When 2 μL of a sample at the lowest concentration (5 pg/μL) is loaded in a reaction, the well contains approximately 1.5 diploid human genome equivalents [19]. Table 1 shows the serial dilution of the control DNA used twice in the experiment. 2 µL of each sample (to be dosed) was added in 96-well plates.

### 2.3. Using Quantifiler™ Trio to Quantify Dilutions of DNA Standards

The initial validation of the qPCR was carried out by analysing the control DNA available with the Quantifiler™ Trio kit. Ten-fold dilution series with 5 concentration points were used, as shown in Table 1. The standard curve was created using serial two-fold dilutions, and quantitative evaluations were carried out using mean values. For each point of the curve, the same dilutions of the standards were assayed, by using them as if they were samples. For each point on the curve, the examination was conducted with five replicates. The synthetic internal PCR control (IPC) value for these samples was also determined.

### 2.4. Reanalysis of the QuantiFiler™ Trio

Twenty different experiments performed to construct the standard curve were assessed to establish the minimum and maximum values for the slope, Y-intercept, and R^2^ for each of the probes used. The threshold cycles of the IPC were also observed to determine if the extraction method used ensured sufficient purification of the traces. 

For an accurate validation of the system applicable to the internal ISO/IEC 17025 method, 757 samples from 20 independent experiments conducted in the period 2016–2024 were used. These samples all came from real forensic cases and included traces of blood, saliva, semen, epithelial cells, bone fragments, and tissues.

#### Standard Versus Sample/Sample Versus Standard

To verify if there were differences between the two replicates used in each of the twenty experiments for constructing the standard curves, the standards were reciprocally used as standards or samples. This was made possible because each experiment could be reanalysed using Design & Analysis Software ver. 2.8.0 (Thermo Fisher Scientific™, USA). We designated each of the two standards as “Sample 1” and “Sample 2” when the other replicate was used as the standard. To assess the differences between the two analyses, we employed the Bland–Altman plot [20]. A Bland–Altman plot is used to assess the measurement difference between two different measurement techniques. In fact, if the measurement of differences is normally distributed and the mean difference is statistically different from zero (i.e., mean ± confidence interval does not contain zero), then we can assert that the two measurement techniques are statistically different. The normality of the dataset was assessed using the Kolmogorov–Smirnov test [21].

### 2.5. Concentration of Extracts from Forensic Samples

In a further experiment, ten DNA extracts from forensic samples taken from subungual grooves of a known individual that had yielded results < 0.003 ng/μL in real-time quantification were concentrated using the Savant SpeedVac™ Vacuum instrument (Thermo Fisher Scientific™, USA). The samples were dried at room temperature and then diluted in TE buffer to 5 μL, suspending them for 30 min at 37 °C. This way, the DNA extracts were concentrated nearly eight-fold compared to the original extracts. No further quantification was performed on these samples to avoid consuming additional samples.

### 2.6. Amplification

For the amplification phase, we validated the PCRs with half the dose. Thus, for both the Globalfiler^TM^ PCR Amplification kit and the PowerPlex^®^ Fusion 6C, the maximum volume we can use is 7.5 μL of extracted DNA. When determining the Y-chromosome profile, especially in cases of sexual assault, we utilise the kit PowerPlex^®^ Y23. In this case, we can use a larger volume, up to 8.75 μL of extracted DNA. Therefore, at least theoretically, the minimum quantity for an analysis with a low-template scenario for autosomal markers is at least 22.5 pg/PCR, and for an analysis with a standard protocol, it is 225 pg/PCR. If an amplification for the Y haplotype is conducted, the quantities are at least 26.5 pg/PCR and 265 pg/PCR for the low-copy-number and standard protocols, respectively. Finally, the concentrated samples were fully amplified using the GlobalFiler^TM^ kit under standard conditions [22].

## 3. Results

### 3.1. Extraction Method

**Figure 2:**
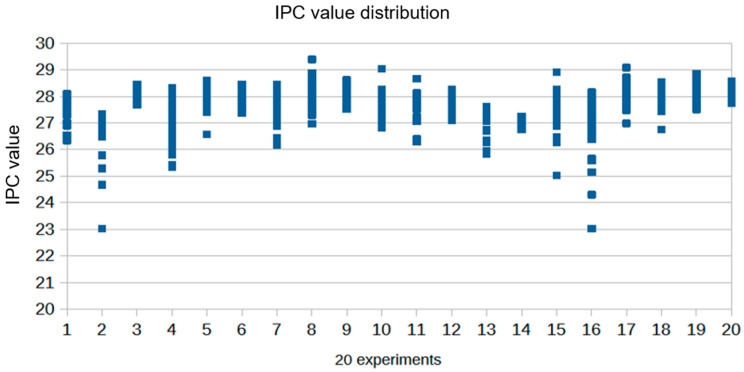
IPC value in real experiments on casework samples. Complete inhibition was observed in only 2 out of 757 DNA samples. The synthetic internal PCR control (IPC) template DNA is present at a consistent concentration across all reactions on a plate. Therefore, the IPC Ct should be relatively constant in typical reactions if PCR inhibitors and/or higher concentrations of DNA are not present in the extract. The use of the IPC system helps us distinguish between true negative sample results and reactions affected by the presence of PCR inhibitors, the assay setup, or a chemistry or instrument failure.

Figure 2 shows the IPC value graph for twenty experiments containing 757 forensic samples of various types. The values are generally distributed closely together and rarely exceed cycle threshold 29, observed in the standard sample showing the highest inhibition (50 ng/μL). Complete inhibition was observed in only 2 samples out of the entire set of forensic samples examined (2/757 undetermined). The quality of the extracts evaluated on a large number of samples showed a reduced quantity of inhibitors in a few samples (2/757). This is largely due to the technology adopted for DNA extraction. Magnetic particle technology, which combines the speed and efficiency of silica-based DNA purification, is fully automated and has effectively ensured the complete removal of inhibitors from treated samples. The particles are ultimately separated from the lysates using a magnet, and even if some particles remain in the tube, they do not act as inhibitors. DNA is then efficiently washed and eluted in the TE buffer, ensuring the applicability of subsequent steps.

### 3.2. Performance of the Quantifiler™ Trio Kit

**Figure 3:**
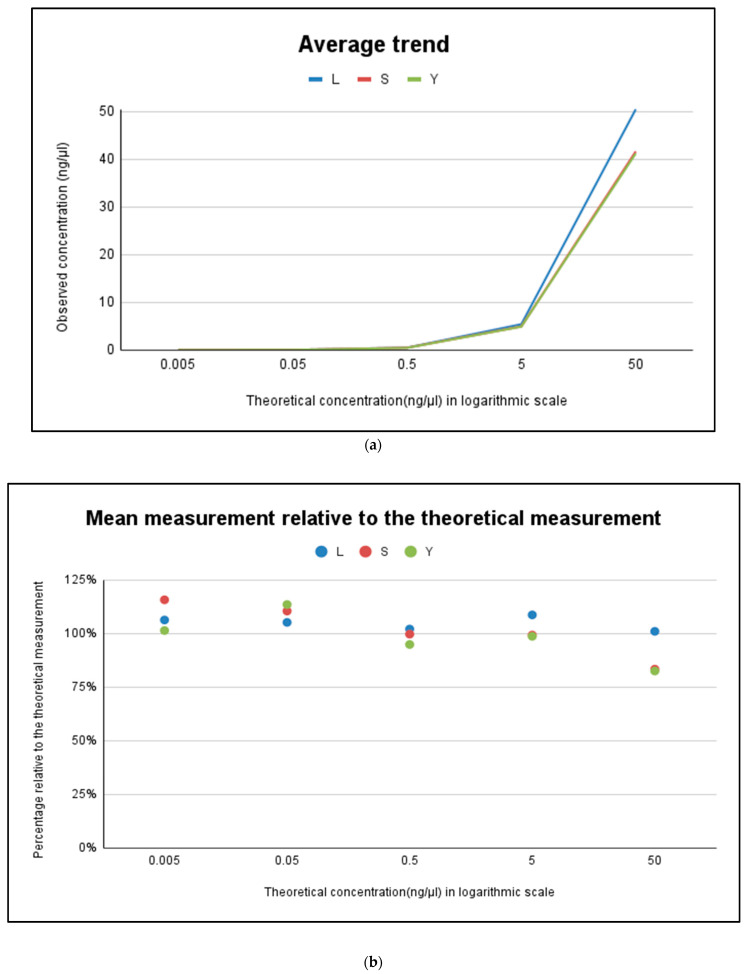
(**a**) Linearity for the standard curve for the Quantifiler™ HP and Trio Kit is from 5 pg/μL to 100 ng/μL as declared by the manufacturer. Correlation between expected and observed quantification of DNA control. (**b**) Percentage correlation between expected and observed quantification of DNA control. Each point on the graph was obtained from the average quantification value of the five points used for constructing the standard curve in a typical quantification reaction (50, 5, 0.5, 0.05, and 0.005 ng/µL) measured in quintuplicate. Linearity for the standard curve for the Quantifiler™ HP and Trio Kit is from 5 pg/μL to 100 ng/μL as declared by the manufacturer.

Table 2 and Figure 3a show the results obtained from the analysis of the five replicates of each standard. The average values of the five measurements were calculated, along with their standard deviations and the differences from the theoretical dosage of each standard. For the higher concentration points of the curve (50 ng/µL), the large probe returns values that are slightly different from the expected value (+1.008%), while the small and Y probes underestimate the value significantly (about −16% and −17%). When dosing highly concentrated samples, it is therefore this probe that provides the most reliable results. From the next point (5 ng/µL) onwards, the large probe proves to be less efficient than the others, overestimating the actual sample (+8.6%). The best match observed among the three probes is for the value of 0.5 ng/µL, where the absolute difference is minimal (Figure 3b). For the lower points, 0.05 and 0.005 ng/µL, all the probes slightly overestimate the expected theoretical values. Figure 3b shows the average measurement relative to the expected theoretical values in comparison to the theoretical linearity values declared by the manufacturer. The IPC probe values remained practically constant across all standard dilutions, within a Ct range of 27.6 to 27.8. We did not observe any increase in Ct values, so the value of 27.7 (average) can be considered the threshold cycle at which a sample shows no inhibition due to excessive DNA concentration or the presence of inhibitors, for the experiments conducted in our laboratory.

**Figure 4:**
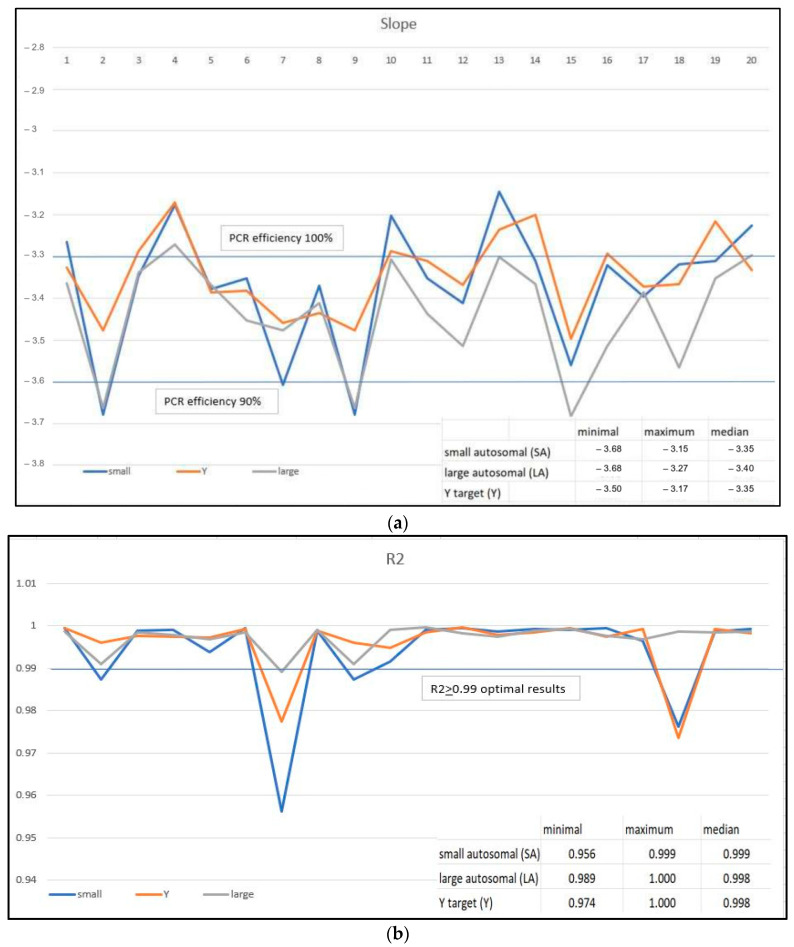

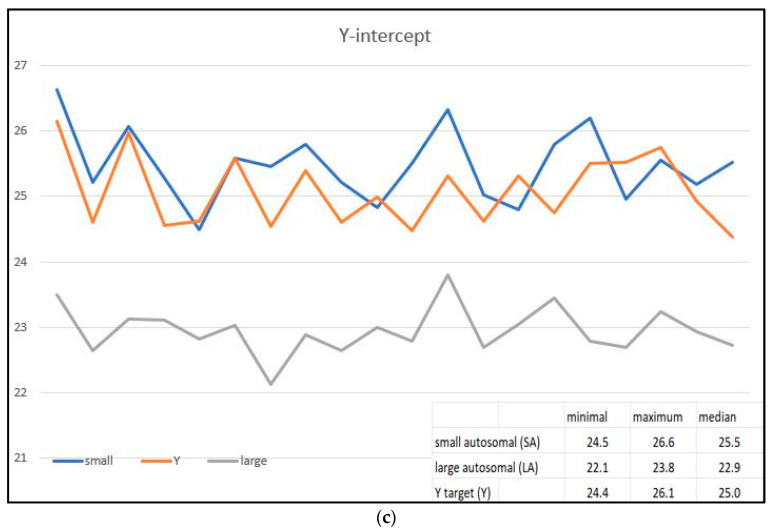
(**a**) The standard curve is a graph of the Ct of quantification standard reactions plotted against the starting quantity of the standards. The software calculates the regression line by calculating the best fit with the quantification standard data points. The regression line formula has the form Ct = m [log (Qty)] + b, where m is the slope, b is the Y–intercept, and Qty is the starting DNA quantity. The slope indicates the PCR amplification efficiency for the assay. A slope of 3.3 indicates 100% amplification efficiency. The figure shows the slopes determined in twenty different experiments. (**b**) R^2^ value—measure of the closeness of fit between the standard curve regression line and the individual Ct data points of quantification standard reactions. A value of 1.00 indicates a perfect fit between the regression line and the data points. The figure shows the R^2^ observed in twenty different experiments. (**c**) The Y–intercept indicates the expected Ct value for a sample with Qty = 1 (for example, 1 ng/μL). The figure shows the Y-intercept results in twenty different experiments for small, Y, and large probes.

Figure 4a–c show the results for the slope, Y-intercept, and R^2^. For the calculation of the linear regression, the five points of the standards have always been used. The examination of the experimental parameters highlighted the following. A slope close to −3.3 indicates optimal, 100% PCR amplification efficiency. In the revalidation of the system, the examination of twenty different experiments regarding the standard curves showed an average slope value of −3.3 for the small and Y probes, and −3.4 for the large probe. The maximum values observed in individual experiments were −3.68 for both small and large probes, while the minimum was −3.15 for a single experiment for the small probe. The Y-intercept value indicates the expected Ct value for a sample with Qty = 1 (for example, 1 ng/µL). Each laboratory should experimentally evaluate the Y-intercept values over time. In addition to variations that can be caused by pipetting of standards or minor lot-to-lot variations in the kits, the Y-intercept can also be affected by instrument-to-instrument variation. As expected, the Y-intercept for the Large Autosomal target is typically lower (mean value 23.0) than the Y-intercept for the Small Autosomal target (25.5) or the Y target (25.1). This is because of the higher copy number of the Large Autosomal target relative to the copy number of the Small Autosomal and Y targets (17). An R^2^ value ≥ 0.99 indicates a close fit between the standard curve regression line and the individual Ct data points of the quantification standard reaction. In the twenty different experiments, the mean values for the three probes were always >0.99% (SA = 0.994, LA = 0.997, Y = 0.996) (Figure 4). As shown in Figure 4b, in two experiments, we observed an R^2^ < 0.98, which was actually due to different outcomes of the preparation of their respective standard pairs. From our experience, this parameter can indeed indicate a significant difference in the quantification results of the samples used for standard preparation.

### 3.3. Standard Versus Sample/Sample Versus Standard Results

**Figure 5:**
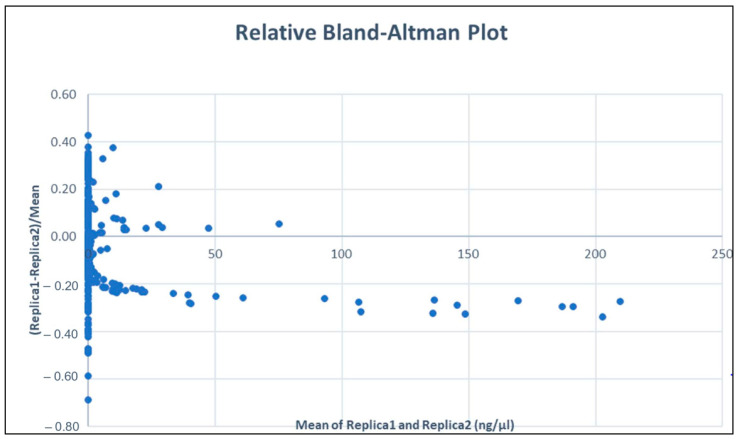
The relative Bland–Altman plot. The mean of the relatives’ differences is (–0.02 ± 0.02).

**Figure 6:**
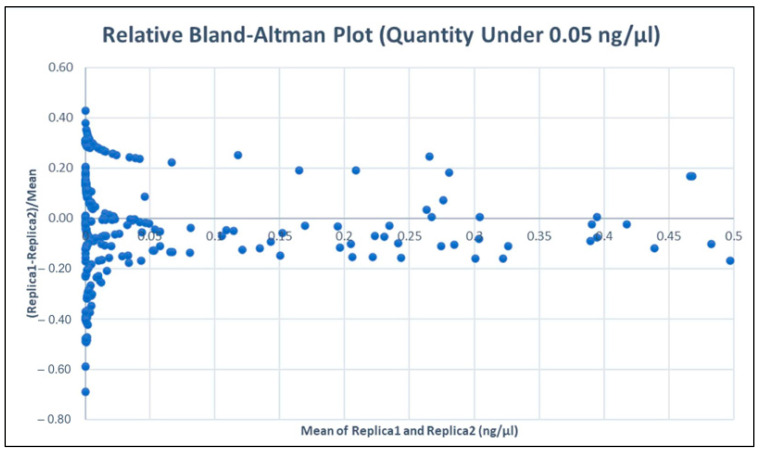
The relative Bland–Altman plot for quantity values lower than 0.05 ng/μL. The mean of the relative difference for these values is 0.02 with a confidence interval of 0.02.

We plotted the standard measurements on the x-axis and the relative difference on the y-axis. We calculated the mean of the relative differences, which was found to be slightly below 0 for all measures but heavily dependent on the very high values. In this paper, we were interested in analysing what happens with low concentration values. Therefore, we repeated the Bland–Altman plot for values below the threshold of 0.05 ng/µL. In this second approach, we found a non-significant difference between the two results (Sample 1 and Sample 2). To be more accurate, we also performed a normality test to verify the normality of the distribution of the relative differences (the y–axis values). As we can see in Figure 5, the measures of Sample 1 for concentrations above 50 ng/µL are lower than those of Sample 2. In fact, if we consider all the concentrations, we find that Sample 1 measures about 20% less compared to Sample 2. However, as shown in Figure 6, if we restrict ourselves to lower concentrations, we find that the two measurements are not significantly different.

So, even though using two standards at the same concentration might theoretically decrease pipetting differences, these measurements do not show significant discrepancies (at least for values lower than 0.05 ng/µL). Therefore, we observe differences between higher and lower values as shown in Figure 5. Consequently, we calculated the mean and confidence interval of the latter (95% confidence interval is calculated as mean ± 1.96 *Standard ErrorWe found that the confidence interval of the mean does not include zero. This indicates that there are significant differences between the actual measurements techniques. However, when focusing on values within our range of interest (values below 0.05 ng/µL) and calculating the mean and confidence interval, we obtain a relative difference of 0.02 ± 0.02; thus, 0 is included, and therefore, no statistically significant differences exist between the two approaches within the range of 0 ng/µL to 0.05 ng/µL. Among the requirements of the Bland–Altman test is that the distribution of the differences should be normal; for this reason, we assessed the normality of the dataset using the Kolmogorov–Smirnov and Lilliefors tests, which indicated that our dataset is sufficiently similar to a normal distribution to be considered as such (see Figures S1 and S2 in the Supplementary Materials). 

### 3.4. Genotyping of Concentrated Samples

**Figure 7:**
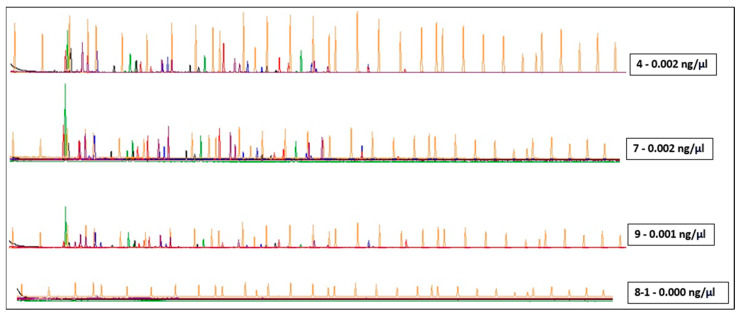
The Figure shows raw data amplification results obtained by using DNA samples from casework obtained from subungual grooves from a known individual. Each sample was obtained by rubbing a swab into the subungual sulcus. 4 = left ring finger; 7 = right index finger; 8-1 = medium right; 9 = right ring finger. The concentration indicated in the boxes for each sample was that before the concentration phase.

**Figure 8:**
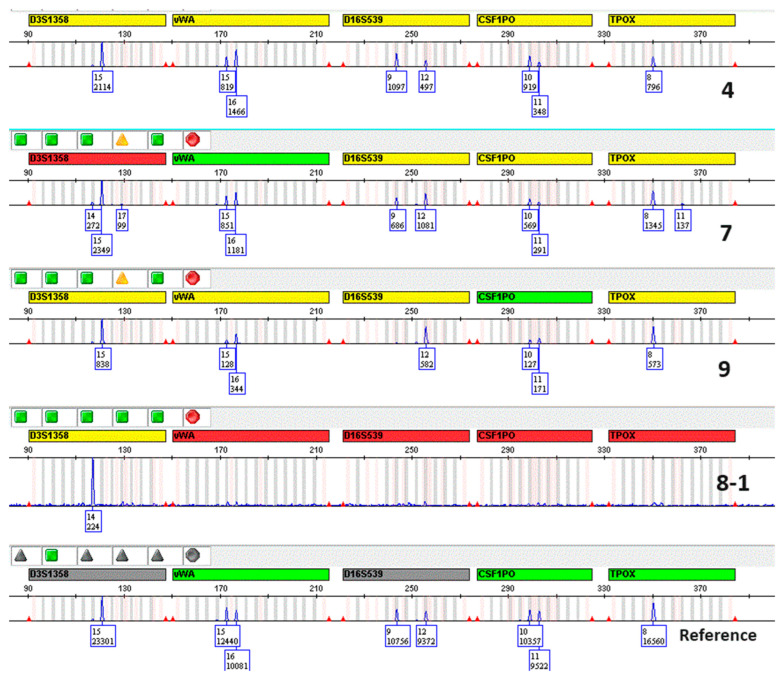
Amplification results using Globalfiler™ PCR Amplification kit. The figure shows the electropherograms for the same samples as in Figure 7, compared to the reference DNA from a saliva sample of the donor (only the blue channel is shown). Although some “drop-in” and “drop-out” phenomena were observed in some loci, the donor’s genetic profile is evident for almost all DNA markers.

DNA extracts from samples that would not have been further examined were instead PCR-amplified after being concentrated approximately 8-fold. Figure 7 shows the results of the electropherograms, demonstrating the presence of amplification products in samples with theoretical concentrations of 0.002 and 0.001 ng/μL. Comparison with the genetic profile of the donors confirmed the correct typing, with the presence of drop-in and drop-out phenomena as expected (Figure 8). A sample with a concentration of 0.000 ng/μL did not yield any typing results, except for a single allele at D3S1358. Although these are preliminary results from a few samples, this method indicates concrete possibilities for successfully determining genetic profiles even from samples containing very few cells which may not be detected by a quantitative test.

## 4. Discussion

Our service primarily focuses on genetic diagnoses in the prenatal field. However, due to the lack of law enforcement laboratories in the Tuscany region (Central Italy), we established a forensic genetics section in 2009, with an ISO/IEC 17025-accredited test since 2012. The laboratory serves as a reference point for forensic genetics in the Tuscany region [23]. As of today, there are another eight highly specialised Italian laboratories, not belonging to law enforcement, located in other Italian regions, which produce genetic profiles accredited according to ISO standards. The purpose of our service is to strive for the highest possible outcome for genetic profile determinations from items related to crimes. The ISO/IEC 17025 accreditation issued by the Italian national accreditation body, Accredia, indeed allows our laboratory to submit genetic profiles to the National DNA Database, managed by the Ministry of the Interior in Rome [24].

The accredited method requires a critical and constant review of the results obtained in the execution of test. We are aware that to achieve accuracy in DNA quantification, Sources of error (e.g., inaccurate measurements) must be minimized must be minimised. Using real-time methodology, we can ensure the detection of human DNA, the identification of male components, and the presence of any inhibitors that interfere with the PCR phase.

Precision and accuracy are therefore critical aspects of DNA quantification in forensic science. We are aware that in the use of manual methods, especially in the pipetting operations of standards and samples to be quantified, random errors can occur. It is known that real-time PCR tests are extremely sensitive, and the detection of Ct values > 35 may indicate the presence of extremely low quantities of DNA. In fact, it is possible to detect Ct values < 40 for extraction control samples and negative control samples while performing real-time PCR with the QuantiFiler™ Trio DNA quantification kit. The detection of such a low quantity of DNA can vary from amplification to amplification based on stochastic effects. Therefore, it is recommended that each laboratory establish a Ct value above which a positive result represents only a background signal [16]. From our experience, we have verified the utility of conducting a thorough re-evaluation of the results obtained from numerous real-time experiments.

We must also acknowledge that in none of the 240 experiments did we ever need to repeat the procedure. Once the real-time reaction has been conducted, even if certain parameters were set incorrectly (such as standards inserted as samples, incorrect quantities in controls, etc.), it is always feasible to reassess the data using the software integrated into the analytical system. Meanwhile, we have extensively re-evaluated the parameters relating to the construction of the standard curve in twenty independent experiments, obtained with five serial dilutions of control DNA used in duplicate. Among the values of R^2^, slope, and Y-intercept, the first is certainly the one that appears the most relevant. R^2^ measures the closeness of fit between the standard curve regression line and the individual Ct data points of quantification standard reactions. If this value is below 0.99, we must consider the quantification results, especially the minimum ones, with an additional margin of uncertainty. 

Regarding high sample concentrations (between 5 and 50 ng/µL or more), we know that quantification results should rely on the results of the broad human probe, as both the small and Y (if it is male DNA) probes provide underestimated results. However, introducing an incorrect aliquot of extracted DNA into an experiment causes limited damage; at most, it involves re-amplifying the sample, which nevertheless remains available in large quantities. The best performance result among the three probes is for the concentration of 0.5 ng/µL. This limit is particularly important for our laboratory because it is very close to the quantitative decision criterion we adopt (0.030 ng/µL) to proceed with amplification according to the standard or low-copy-number method (Figure 1).

The most serious issues can occur, instead, in the failure to generate useful genetic profiles from low-concentration samples.

The QuantiFiler™ Trio DNA quantification kit can detect DNA concentrations < 5 pg/μL; however, at concentrations < 5 pg/μL, stochastic effects or the statistical effect of random sampling of alleles present in a very low number of copies can produce significant variability in assay results. When using samples containing DNA in this concentration range, replicate analyses are indeed necessary to confirm the true absence of DNA. In the review of this phase of the process, it was possible to define more accurate limits based on the quantification results for subsequent choices regarding the possible determination of genetic profiles. However, the relative Bland–Altman plot in our area of interest did not show significantly relevant differences, with an average ratio of 0.02 ± 0.02 (<(x1 − x2)/(x1 + x2)/2 >= 0.02 ± 0.02), meaning that, on average, one of the two measurements differed within a range of approximately 0% to 4% from the other. Therefore, we have decided to adjust the limits set by the initial validation for the method (Figure 1) based on the results from the evaluations described in this article. Considering the deviations observed in the quantification of the standards (Table 2 and Figure 3b) and the results of the Altman test (Figure 5 and Figure 6), we consider it necessary to use less strict limits for our choices regarding those currently used for the application of the standard and low-copy-number protocols. This allows us to make better choices by optimising the conduct of the test, pending a more effective implementation of the method. 

Among the actions we plan to undertake, certainly, the introduction of an automated method for the preparation of quantification plates could reduce random errors in the pipetting of standards and samples during the construction of the calibration curve and in the retrieval of samples. The method could be further improved by using a reduced PCR reaction volume, as demonstrated by some authors [25]. This approach could also be applied to the quantification phase itself, halving both the reagents and the input extract to be measured. This would enhance the functionality of quantifying extracts from forensic samples and significantly reduce the costs of the entire analytical process.

However, there are even more substantial improvements that we aim to systematically verify shortly. The concept of usability derives directly from the indications of the Italian DNA Database, for which a profile can be included for national comparisons if at least seven genetic markers, including amelogenin, are correctly typed. However, to be transmitted worldwide through the European Network of Forensic Science Institutes (ENFSI), the number of markers must be at least ten.

Naturally, this new sample analysis method will require a careful validation process. A possible development could foresee, after observing quantities < 0.002 ng/μL, an initial PCR cycle, followed by verifying the quality of the generated genetic profile. If the result shows an acceptable profile, a second PCR could be performed to generate an additional profile and thus determine a consensus profile. If the result is not usable, the sample could be concentrated and then amplified from the extract suspended in a minimal amount of TE buffer. However, in this way, almost half of the extract would be used for the initial analyses, and such a structured activity has little practical application in our laboratory, requiring too many analytical sessions for a single forensic case.

An alternative choice could be using sample concentrations < 0.002 ng/μL to apply a single PCR reaction without further quantification, as we have verified in some samples obtained from scrapings under the nails of a known donor, which has demonstrated the possibility of generating genetic profiles suitable for comparisons. However, this method is not without risks, as it involves a single amplification reaction, the outcome of which cannot be verified by a second independent one. It will require thorough verification before it can potentially be applied in routine practice.

## 5. Conclusions

This study is based on a decade of experience in forensic sample analysis at the laboratory designated by the Tuscany Region as a reference point for forensic genetics. The mandatory validation processes in the accreditation context have been useful for a critical review of any part of the test process. We aimed to revalidate the quantification phase, which precedes the decision on whether a trace extract should undergo typing analysis or not. This critical review has highlighted that the uncertainty due to the technical phases of quantification preparation requires the decision limits for the subsequent phases of the analytical process to be redefined. Moreover, this study also emphasises another aspect that will require further, in-depth validation before being applied for the analysis of real samples. By applying a concentration step to some extracts and subjecting the sample to a single PCR phase, usable genetic profiles for comparisons were generated. It is therefore evident that the latter analytical phase must be included in the process before truly considering a trace as unsuitable for generating a genetic profile for identification purposes. It is evident that there are still opportunities to improve this critical analytical phase, which is perfectly aligned with the expectation of continuous improvement as indicated by the ISO/IEC 17025 standard.

The vast majority of laboratories use manual methods to quantify forensic extracts, and thus, this work should help in paying attention to the execution of this phase. We believe that the critical review of quantifications reported in this study can contribute to an improvement in the entire forensic analysis process, emphasising how to take into account the uncertainty always present in any laboratory activity and, ultimately, the consistency of human activities. 

## Figures and Tables

**Table 1:** Quantifiler™ THP DNA Standard stock diluted in Quantifiler™ THP DNA Dilution Buffer (THP) was used for the construction of the standard curve. A minimum input volume of 10 μL DNA for dilutions was used to ensure the accuracy of manual pipetting, as suggested by the Quantifiler™ Manual. The concentration ranged from 50 ng/μL (St. 1) to 0.005 ng/μL (St. 5).

Standard	Concentration (ng/µL)	Volume	Dilution Factor
**St. 1**	50.000	10 µL [100 ng/µL stock] + 10 µL QuantiFiler™ THP DNA dilution buffer	2X
**St. 2**	5.000	10 µL [Std. 1] + 90 µL QuantiFiler™ THP DNA dilution buffer	10X
**St. 3**	0.500	10 µL [Std. 2] + 90 µL QuantiFiler™ THP DNA dilution buffer	10X
**St. 4**	0.050	10 µL [Std. 3] + 90 µL QuantiFiler™ THP DNA dilution buffer	10X
**St. 5**	0.005	10 µL [Std. 4] + 90 µL QuantiFiler™ THP DNA dilution buffer	10X

**Table 2:** Experiment with DNA controls dosed like samples. Each dilution of the control DNA mixture used to construct the curve was assayed five-fold as if it were an unknown sample. The average values of the measurements, the standard deviation, and the percentage difference with respect to the respective theoretical points were calculated. The small human autosomal probe (Small) consists of diploid multi-copies of 80 bp, the large human autosomal probe (Large) of diploid multi-copies of 214 bp, the human male target (Y) of haploid multi-copies of 75 bp, and the synthetic IPC probe of 130 bp. The whole data of the experiment are available in Table S1.

		Theoretical Input in ng/µL				
		50	5	0.5	0.05	0.005
**probe**						
**Large autosomal**	average	50.504	5.435	0.510	0.053	0.005
	standard deviation	5.503	0.227	0.028	0.003	0.001
	% difference (BIAS)	1.008	8.692	2.077	5.188	6.312
**Small autosomal**	average	41.664	4.963	0.498	0.055	0.006
	standard deviation	13.068	0.318	0.090	0.003	0.001
	% difference (BIAS)	−16.672	−0.730	−0.309	10.492	15.734
**Y**	average	41.237	4.934	0.474	0.057	0.005
	standard deviation	7.154	0.517	0.062	0.009	0.001
	% difference (BIAS)	−17.526	−1.321	−5.145	13.536	1.384

## Data Availability

The data presented in this study are available on request from the corresponding author. The data are not publicly available due to privacy.

## Supplementary Materials

The following supporting information can be downloaded at https://www.mdpi.com/article/10.3390/genes15060759/s1: Table S1: Experiment results with DNA controls dosed like samples. Each dilution of the control DNA mixture used to construct the curve was assayed in triplicate as if it were an unknown sample. Sample setup, amplification data, results, and final calculations included IPC values are shown; Figure S1: Report of the normality test. Figure S2: The histogram of the relative differences in the Bland-Altman plot.
