# Supplementary figures and images for: Looking into the Quantification of Forensic Samples with Real-Time PCR
Source: Genes (Basel). 2024 Jun 9;15(6):759. doi: 10.3390/genes15060759 (PMC11202440; doi:10.3390/genes15060759)

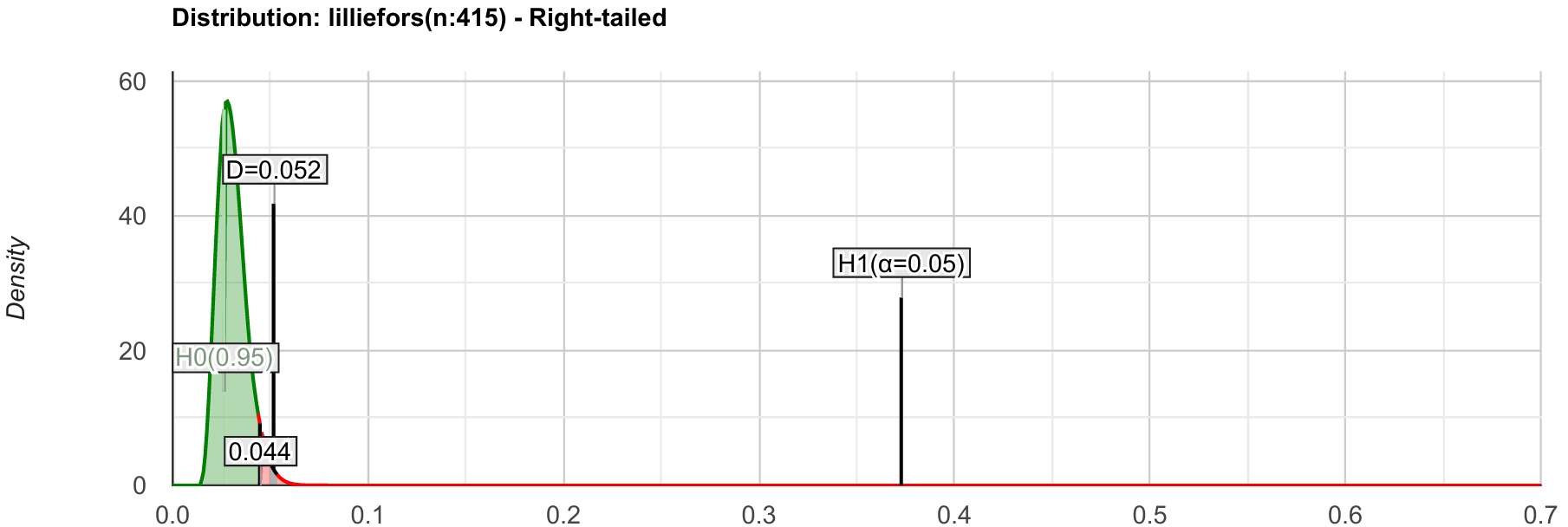

Supplement: Supplementary file 1 [file genes-15-00759-s001.zip › Figure S1.jpeg]

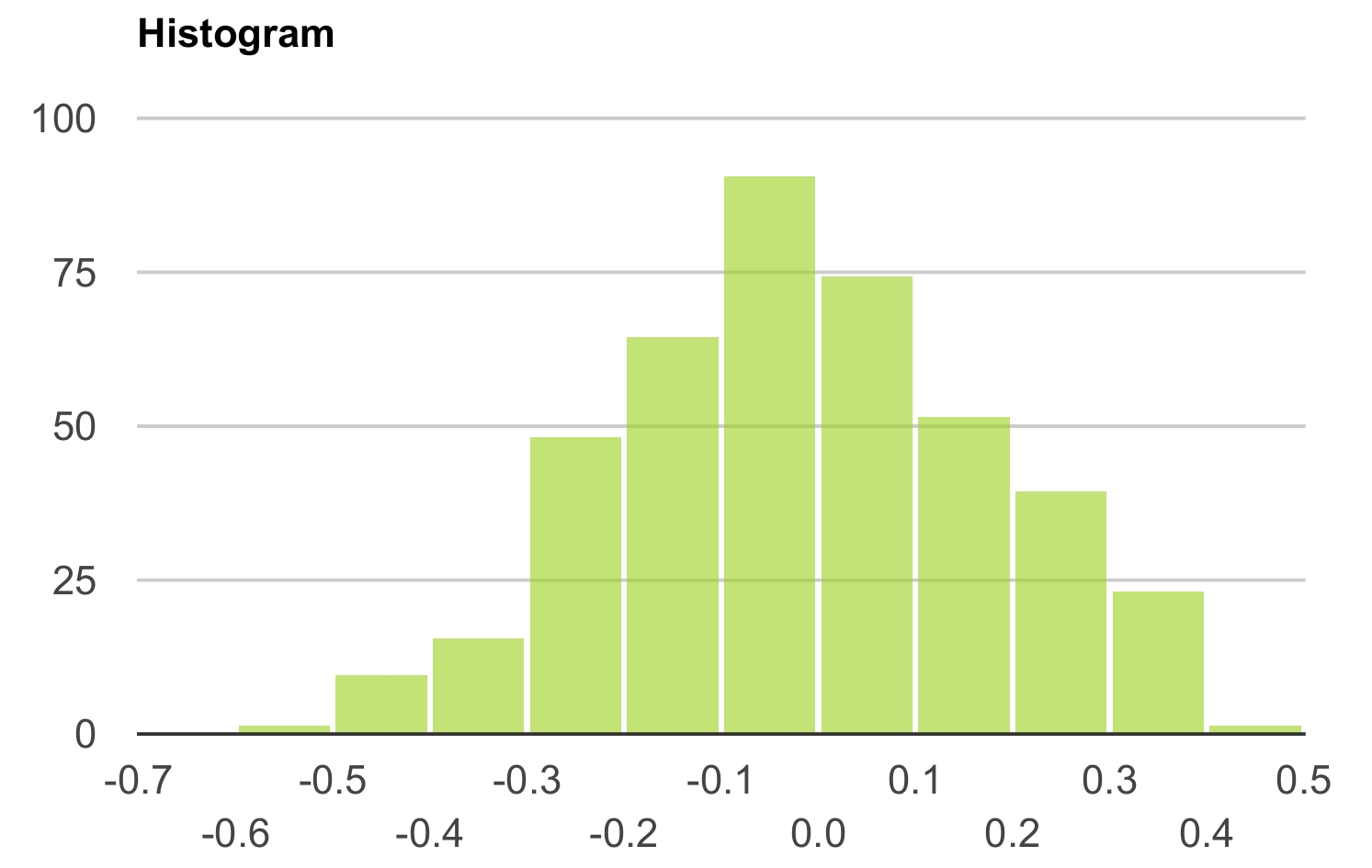

Supplement: Supplementary file 1 [file genes-15-00759-s001.zip › Figure S2.jpeg]
